# Novel metallic implantation technique for osteochondral defects of the medial talar dome: A cadaver study

**DOI:** 10.3109/17453674.2010.492764

**Published:** 2010-07-16

**Authors:** Christiaan J A van Bergen, Maartje Zengerink, Leendert Blankevoort, Maayke N van Sterkenburg, Jakob van Oldenrijk, C Niek van Dijk

**Affiliations:** Orthopedic Research Center Amsterdam, Department of Orthopedic Surgery, Academic Medical Center, University of Amsterdamthe Netherlands

## Abstract

**Background and purpose:**

A metallic inlay implant (HemiCAP) with 15 offset sizes has been developed for the treatment of localized osteochondral defects of the medial talar dome. The aim of this study was to test the following hypotheses: (1) a matching offset size is available for each talus, (2) the prosthetic device can be reproducibly implanted slightly recessed in relation to the talar cartilage level, and (3) with this implantation level, excessive contact pressures on the opposite tibial cartilage are avoided.

**Methods:**

The prosthetic device was implanted in 11 intact fresh-frozen human cadaver ankles, aiming its surface 0.5 mm below cartilage level. The implantation level was measured at 4 margins of each implant. Intraarticular contact pressures were measured before and after implantation, with compressive forces of 1,000–2,000 N and the ankle joint in plantigrade position, 10° dorsiflexion, and 14° plantar flexion.

**Results:**

There was a matching offset size available for each specimen. The mean implantation level was 0.45 (SD 0.18) mm below the cartilage surface. The defect area accounted for a median of 3% (0.02–18) of the total ankle contact pressure before implantation. This was reduced to 0.1% (0.02–13) after prosthetic implantation.

**Interpretation:**

These results suggest that the implant can be applied clinically in a safe way, with appropriate offset sizes for various talar domes and without excessive pressure on the opposite cartilage.

## Introduction

Osteochondral talar defects (ODs) have been reported in 7–41% of patients with lateral ankle ligament rupture (Lippert et al. 1989, van Dijk et al. 1996, Takao et al. 2003). Two-thirds of talar ODs are located on the medial talar dome; these lesions are often deep and cup-shaped and are generally larger than lateral lesions (Raikin et al. 2007). ODs may develop into subchondral cysts (Jackson et al. 2001, van Dijk et al. 2010). This is thought to be due to intrusion of fluid through a bony defect, and hampers effective treatment (Scranton and McDermott 2001, Robinson et al. 2003, Reilingh et al. 2009, van Dijk et al. 2010).

The primary treatment of most ODs is arthroscopic debridement and microfracturing, with a success rate of 85% (van Dijk and van Bergen 2008, Zengerink et al. 2009). For failed primary treatment and large defects, there are various alternative treatment options, including cancellous bone grafting, osteochondral autograft transfer, and autologous chondrocyte implantation (van Bergen et al. 2008, Zengerink et al. 2009). However, each of these has disadvantages such as donor site morbidity, limited availability, and two-stage surgery (van Bergen et al. 2008, Paul et al. 2009).

**Figure 1.**
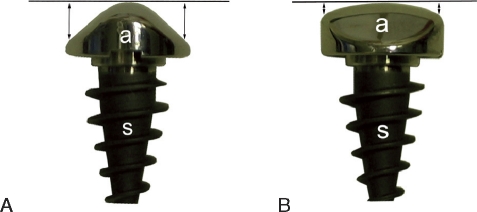
The metallic implant consists of a screw (s) and an articular component (a) with a diameter of 15 mm. There are 15 articular components with a variety of offset sizes (↕) with steps of 0.5 mm relative to the center. A. Anterior-posterior view: in this plane offset sizes range from 3.5 to 5.5 mm. B. Medial-lateral view, with offset sizes ranging from 0.5 to 2.0 mm.

A novel metal inlay implantation technique (HemiCAP) was developed in 2007 for medial defects after failed primary treatment (Figure 1). Its clinical goals are to reduce pain and prevent (further) cyst formation by resurfacing the OD. The talar implant set consists of 15 offset sizes to provide an implant that matches the geometry of the medial talar dome in a variety of talar specimens. A precise surgical technique is required in terms of implantation depth, position, and orientation because of the biomechanical properties of the ankle joint. A protruding implant may damage the opposite cartilage by causing excessive contact pressures during loading, which is thought to be due to “plowing” of the cartilage (Loening et al. 2000, Milentijevic and Torzilli 2005, Custers et al. 2007). On the other hand, a deep implant might result in collapse of the adjacent cartilage due to insufficient support (Jackson et al. 2001). Hence, an optimum middle course is aimed for. The talar dome cartilage thickness was found to be 1.42 (SD 0.18) mm in an in vivo study (Wan et al. 2008). Talar cartilage peak contact strain under weight-bearing conditions reached 35% (SD 7) (Wan et al. 2008). We therefore propose an optimal implantation depth of 0.5 mm (35% of 1.4 mm) below the level of the articular cartilage surface.

The purpose of this study was to test the following hypotheses: (1) the set of 15 offset sizes is appropriate for a variety of talar dome curvatures; (2) the prosthetic device can be reproducibly implanted slightly recessed; and (3) excessive pressure on the opposite tibial cartilage can be prevented with 0.5 mm recessed implantation. To test these hypotheses, the prosthetic devices were implanted in cadaver ankle specimens, the implantation levels were measured, and the intraarticular contact pressures were measured before and after implantation.

## Material and methods

### Specimens

12 fresh-frozen (–20°) cadaver ankles, amputated halfway along the tibia, were obtained from donors. During their lives, the donors had given informed consent for the use of their bodies for scientific or educational purposes. The specimens were thawed at room temperature. Normal ranges of motion and ligament stability were confirmed by physical examination. The talar dome was surgically exposed by a medial malleolar osteotomy. The modified Noyes and Stabler classification, as described by Recht et al., was used to grade macroscopically the talocrural joint cartilage surfaces (Noyes and Stabler 1989, Recht et al. 1996). 11 specimens were graded 0 (intact cartilage). 1 specimen had a grade 3A lesion and was excluded from the study. The remaining 11 ankles (6 left ankles) were from 4 males and 6 females (one bilateral) with a mean age of 79 (66–89) years.

### Implant

The articular prosthetic device (HemiCAP; Arthrosurface Inc., Franklin, MA) consists of a fixation screw and an articular component, which are connected via a taper lock (Figure 1). The cannulated fixation screw is made of titanium to allow osseointegration of the subchondral bone. The articular component is made of a cobalt chrome alloy with titanium spray undercoating, and has a diameter of 15 mm. There are 15 offset sizes, with increment sizes of 0.5 mm in the sagittal (0.5–2.0 mm) and coronal (3.5–5.5 mm) planes. The development of the offset sizes was based on geometric data reconstructed at our institution, using computed tomography scans of 52 patients with a talar OD (unpublished data).

### Surgical technique

CNvD and 2 assistants performed all surgical procedures according to a standardized technique. A longitudinal skin incision was made, and 2 screw holes were predrilled in the medial malleolus from distal to proximal. An anterior arthrotomy was performed, and the periosteum of the medial malleolus was incised. 2 Hohmann retractors were placed to protect the anterior and posterior vital structures. Using an oscillating motorized saw and a chisel, the surgeon exposed the medial talar dome by creating a medial malleolar osteotomy. The osteotomy was aimed at the intersection between tibial plafond and medial malleolus, as described by Seil et al. (2001). At this stage of the procedure, the pre-implantation contact pressures were measured (see “Pressure measurements” below).

At the second stage of the operation, the prosthetic device was implanted. The lateral malleolus of the specimen was placed on a rolled-up apron in order to evert the talus for better exposure. A Kirschner wire was drilled through the distally retracted medial malleolus into the inferior talar body to maintain good exposure. A guide pin was drilled into the talus, perpendicular to the medial talar trochlea, using a special drill guide. This guide pin ensured good orientation of the cannulated instruments throughout the procedure. The position of the guide pin was initially checked with a contact probe, and it was repositioned in case of suboptimal position. After drilling and tapping of the pilot hole, the fixation screw was inserted into the subchondral bone up to a precise depth, indicated by a mark on the screwdriver at the level of the superficial cartilage. After flushing and cleaning, the implantation level of the screw was verified using a small trial button. If necessary, the screw level was adjusted carefully by turning it clockwise or anticlockwise.

The level of the adjacent cartilage relative to the screw (i.e. the radius of curvature) was measured anteriorly, posteriorly, medially, and laterally using a contact probe that was provided with the implantation set. Subsequently, a 15-mm circular incision of the cartilage was made using a circle-cutter, and a 15-mm implant bed was reamed. The articular component was considered appropriate when its offset sizes corresponded with the surface mapping measurements. An articular sizing trial cap was placed, aiming 0.5 mm below the level of the adjacent cartilage. If satisfied, the surgeon placed the final implant loosely on the screw to confirm correct rotation, and engaged it by means of a gentle hammer-stroke on an impactor. The ankle joint was flushed and cleaned to remove any remaining debris.

### Implantation level

After the tali were excised and removed from the specimens, CJAvB measured the implantation level at the transition of the prosthetic implant and the adjacent cartilage surface at 4 locations (anterior, posterior, medial, and lateral) using a digital calliper in a blinded fashion; the readings were read and recorded by an assistant. The calliper (Absolute Digimatic Calliper 500-series; Mitutoyo Nederland BV, Veenendaal, the Netherlands) had a resolution of 0.01 mm and an accuracy of 0.02 mm (manufacturer's data). Each location was measured twice and averaged for analysis. Whether the central part of the implant protruded was assessed visually, specifically by a view tangential to the articular surface from both anteriorly and medially.

### Testing material

**Figure 2.**
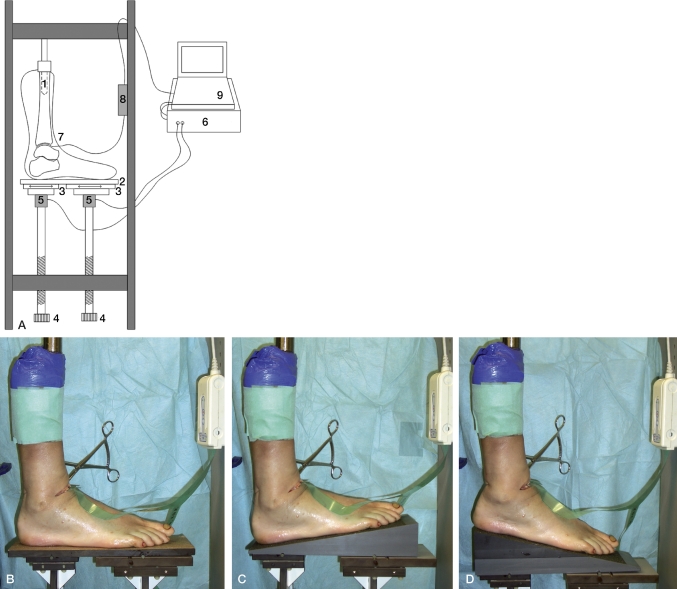
Pressure measurements. A. Schematic drawing of the testing apparatus and data acquisition. The specimen with an intramedullary tibial nail (1) was mounted on a base-plate (2), with a rough surface, on a ball bearing (3). The ball bearing allowed anteroposterior translation (↔) of the foot in plantigrade position, but not in the plantarflexed and dorsiflexed positions. The force was applied by turning two handle-actuated lead screws (4) that gradually moved the base-plate upwards. The force transducers (5) were attached to a data-acquisition system (6) that converted the output from the strain gages into force (N). A Tekscan sensor (7) was placed in the ankle joint and was connected via a USB interface (8) to a computer (9). B. In the testing apparatus, the specimens were loaded with a force of 1,500 N and with the ankle in the neutral position. C. Loading with 2,000 N in 10° dorsiflexion. D. Loading with 1,000 N in 14° plantar flexion.

Contact pressure was measured using a thin film pressure-sensitive system (Tekscan Inc., Boston, MA), which is more accurate in measuring surface area, force, and pressure than the Fuji film pressure-sensitive technique (Bachus et al. 2006). The sensors are 0.1 mm thick and consist of electrical contacts in two perpendicular directions (rows and columns), separated by a semi-conductive layer. This layer provides an electrical resistance at each of the intersecting points. By measuring the changes in electrical current at each intersection point, the pressure distribution pattern can be measured and displayed on a screen. Sensor type 4,000 was used, which has 572 sensing elements on a surface of 28 × 33 mm (62 sensing elements per cm^2)^, and a column and row width of 1.27 mm. The flexible pressure sensor was connected to a computer containing I-scan software (Figure 2).

A mechanical testing apparatus, developed by the Medical Technical Development Department of our institution, loaded the ankle during testing (Figure 2). On top it consisted of a shaft where a tibial nail was inserted; at the base it consisted of a base-plate with a rough surface on a ball bearing. The ball bearing allowed anteroposterior translation of the foot in neutral (plantigrade) position but not in the plantarflexed and dorsiflexed positions. To place and hold the ankle specimens in the desired flexion angle, custom-made wedge supports were attached to the base-plate (0° for plantigrade, 10° for dorsiflexion, and 14° for plantar flexion). These angles correspond to the mean sagittal ankle motion during the stance phase of gait in normal subjects (Stauffer et al. 1977). The wedges were made of polyvinyl chloride and were covered with sandpaper to prevent slipping of the specimens. The force was applied by gradually moving the base-plate upwards by turning 2 handle-actuated lead screws. The force transducers of the apparatus were attached to a data acquisition system that, after calibration, converted the output measured by the strain gages into force.

### Pressure measurements

Intraarticular contact pressures were measured before and after implantation. One particular sensor was used for each specimen to correct for any minor differences between the sensors, and thus to permit reliable comparison of pre- and post-implantation pressures. Every test was performed twice and the average of both tests was used for data analysis.

According to the manufacturer's guidelines, each sensor was conditioned by applying 3 loads of 1,600 N with intervals of 30 s. The sensitivity was adjusted accordingly. The sensors were equilibrated and calibrated before every testing sequence. For equilibration, each sensor was inserted into a uniform pressure applicator (pressurized chamber) that was inflated to 6.5 bar. For calibration, a lever with weights attached was used, which allowed application of known forces to the sensor through a silicone interface. A 2-point calibration method was applied by placing the sensor under the lever, between the metal and silicone parts, and loading it with forces of 750 N and 3,000 N for 30 s, with an interval of 3 min.

A transverse anterior incision was made, and the sensor was inserted in the ankle joint. It covered the talar dome and was sutured to the surrounding soft tissue. Digital photographs of the sensor positioning were taken at pre-implantation testing to verify and ensure the same position during the post-implantation testing. The osteotomy was closed by placing a Weber bone clamp on the distal tibia, and was fixated by inserting 2 malleolar screws in the predrilled holes. The bone clamp was left in place to support the screw fixation during testing. An intramedullary nail was inserted into the tibial shaft, and the specimen was mounted on the testing apparatus.

An axial load was gradually applied for approximately 30 s, held in a stationary position for another 30 s, and then gradually released. After 30 s, a relatively stable condition is reached where changes in cartilage contact deformation and area are negligible (Li et al. 2008). To approximate forces and motion during the stance phase of gait, we applied loads of 1,500 N with the ankle in a neutral position, 2,000 N with the ankle in 10° dorsiflexion, and 1,000 N with the ankle in 14° plantar flexion. These loads represent 1.5 up to 3 times body weight and are within the range of previously reported biomechanical ankle studies (Macko et al. 1991, Christensen et al. 1994, Kura et al. 1998, Millington et al. 2007). Recordings were obtained with a sample frequency of 1 Hz. The data that were acquired 30 s after the maximum load had been reached were used for data analysis.

After the sensor was removed from the specimen and cleaned, it was again loaded under the calibration lever to check its accuracy. In 1 specimen, the sensor proved to be inaccurate; it was replaced and the pressure testing was repeated.

### Data analysis

**Figure 3.**
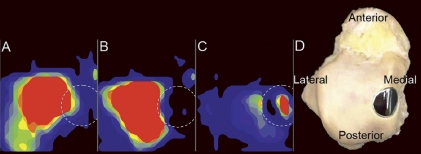
Contact pressures in the ankle joint before and after implantation of the prosthetic device. The circle indicates the position of the implant. The color range represents the spectrum of pressures (high: red; low: blue). A. Contact pressure in a representative specimen (no. 8) in neutral position before implantation. B. Contact pressure in the same specimen in neutral position after implantation. C. Post-implantation contact pressure of the single specimen (no. 11) that showed the peak pressure over the implant (red), in the plantarflexed position. D. An excised talus, viewed from superiorly, demonstrating the position of the implant.

I-scan software version 5.72 was used to qualitatively analyze the Tekscan-recorded pressure measurements. To determine the pressure (MPa) of the implant relative to the total pressure of the sensor, the area of the implant was selected manually. The same area was selected for the pre-implantation measurements. In both measurements, the percentage of pressure over this area was calculated relative to the total pressure of the sensor (Figure 3).

In the case of normal distribution, values are presented as mean (SD) and 95% CI. In the case of skewed distribution, values are presented as median and range.

## Results

**Figure 4.**
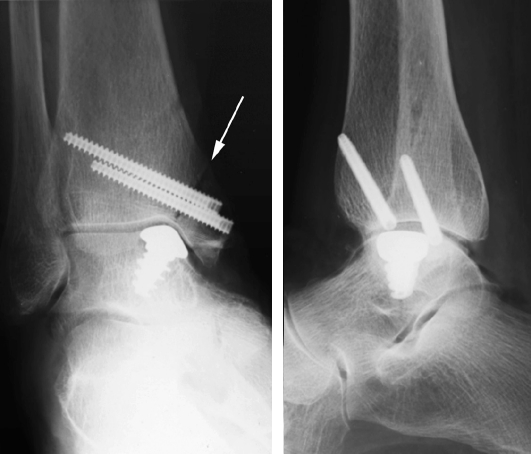
Radiographs of a right specimen (no. 7) with an implant. The arrow indicates the medial malleolar osteotomy fixated with 2 screws.

In all cases there was an appropriate articular component available, i.e. corresponding to the contact probe measurements taken during surgery (Figure 4). 6 of the 15 offset sizes of the articular components were used. The required offset sizes ranged from 0.5 to 1.5 in the sagittal plane and from 3.5 to 5.0 in the coronal plane (Table 1).

**Table 1.** Offset sizes and implantation levels of the implants (in mm). Negative values represent protruding edges

Specimen	Offset size **^a^**		Implantation level				
no.	AP	ML	Anterior	Posterior	Medial	Lateral	Mean **^b^**
1	1.5	5.0	1.07	0.48	-0.32	0.66	0.47
2	1.0	3.5	1.47	0.78	0.28	0.73	0.82
3	1.0	3.5	0.54	0.44	0.33	0.49	0.45
4	1.0	5.0	0.27	0.77	0.36	0.72	0.53
5	1.5	3.5	0.59	0.18	0.24	0.36	0.34
6	0.5	3.5	0.63	0.86	0.19	0.77	0.61
7	1.5	3.5	0.88	0.77	–0.72	0.65	0.40
8	1.0	3.5	0.76	0.17	–0.07	0.41	0.32
9	0.5	3.5	–0.20	0.56	–0.50	0.57	0.11
10	0.5	3.5	0.77	0.62	–0.08	0.29	0.40
11	1.5	4.5	0.45	0.32	0.59	0.59	0.49
Mean **^c^** (SD)			0.66 (0.43)	0.54 (0.25)	0.03 (0.40)	0.57 (0.16)	0.45 (0.18)

**^a^** AP: anterior-posterior plane; ML: medial-lateral plane.

**^b^** Mean of anterior, posterior, medial, and lateral implantation level.

**^c^** Mean of 11 specimens.

**Figure 5.**
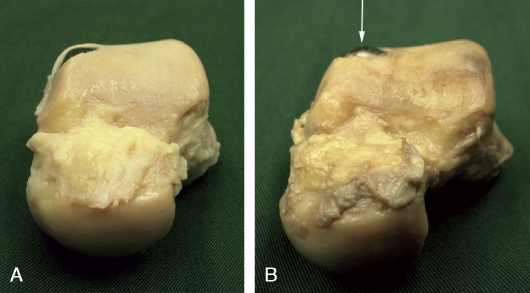
Macroscopic anterior view of the implantation level after excision of the talus. A. Representative specimen (no. 10) showing the implant slightly recessed (n = 10). B. In 1 specimen (no. 11), the implant was protruding at the center (arrow).

The mean implantation level was 0.45 (SD 0.18) mm (95% CI: 0.33–0.57) (Table 1). The lateral part of the implant, located on the talar dome, was measured to be recessed a mean of 0.57 mm (SD 0.16) mm. The medial part of the implant, located on the medial talar facet, was the least recessed (mean 0.03 (SD 0.40) mm). The central part of the implant, as assessed by visual inspection, was recessed in 10 specimens and protruding in 1 specimen (no. 11) (Figure 5).

During the post-implantation pressure measurements, one specimen (no. 2) fractured (comminutive intraarticular distal tibial fracture), and it was excluded from pressure analysis. In 2 specimens (nos. 3 and 11) the pre-implantation contact pressure was not measured, as the screw component had already been implanted during a pilot experiment.

Of the remaining 8 specimens, in all positions combined, the median pressure of the prosthetic area before implantation was 3.3% (0.02–18) relative to the total contact pressure. After implantation, the median prosthetic pressure decreased to 0.09% (0.02–13) in these 8 specimens. The highest median contact pressure was measured in the neutral position and the lowest in the plantarflexed position, both before and after implantation (Table 2).

**Table 2.** Contact pressures of the implant (%) relative to the entire sensor in the three joint positions

Specimen	Neutral		10° dorsiflexion		14° plantar flexion	
no.	pre.	post.	pre.	post.	pre.	post.
1	3.7	0.14	4.1	0.06	0.10	0.10
2 **^a^**	–	–	–	–	–	–
3 **^b^**	–	0.04	–	0.02	–	0.11
4	0.08	0.12	1.7	0.06	0.04	0.12
5	18	13	14	13	11	3.2
6	0.49	0.84	0.55	2.2	2.6	0.04
7	3.0	0.04	0.02	0.02	6.5	0.04
8	5.8	0.08	3.2	0.16	1.7	0.08
9	17	0.26	18	0.21	3.3	0.03
10	6.9	0.02	4.2	0.02	2.2	0.05
11 **^b, c^**	–	13	–	0.92	–	44
Median **^d^**	4.8	0.13	3.7	0.11	2.4	0.07

Pre: before implantation; post: after implantation.

**^a^** Fracture during loading.

**^b^** No measurements before implantation.

**^c^** Protruding central implant.

**^d^** Median values of specimens 1 and 4 through 10.

The protruding implant (no. 11) resulted in an elevated contact pressure in the plantarflexed position (relative pressure: 44%) (Figure 3). In the neutral and dorsiflexed positions, the relative prosthetic pressures of this specimen were 13% and 0.92%, respectively (Table 2).

## Discussion

In this study, the novel metallic inlay implant was investigated before clinical use. The range of available offset sizes was found to be appropriate for the variety of ankle specimens used in this study. The prosthetic device was implanted on average 0.45 mm (95%CI: 0.33–0.57) below the articular cartilage level. With this implantation level, excessive pressures on the opposite tibial cartilage were prevented.

There have been few studies dealing with the level of implantation of metallic implants and its effect on opposing cartilage. In a comparative rabbit study, Custers et al. (2007) showed that protruding implantation of metal plugs in the femoral condyle caused the most damage to the opposite tibial cartilage, and that flush implantation caused the least damage. Their deep implantations were below the level of the subchondral plate and therefore cannot be compared with the average implantation level in our study. Becher et al. (2008) compared implantation levels of a femoral condyle HemiCAP using human cadaver specimens. They concluded that protruding implantation led to substantially increased peak contact pressure, as also occurred in our single protruding implantation (specimen no. 11). However, the knee and ankle cannot be compared because the ankle joint is more congruent and its cartilage has different mechanical and biochemical properties (Shepherd and Seedhom 1999, Treppo et al. 2000).

The protruding central part of the implant in specimen no. 11 was due to an elevated screw rather than an inappropriate articular component. The level of the screw determines the central height, while the offset sizes of the articular component determine the peripheral height. It is thus crucial to implant the screw to a precise level, which is indicated by a mark on the screwdriver. The level of the implanted screw can be checked initially by placing a trial button, and adjusted if necessary. The availability of higher offset sizes allows for a recessed peripheral implantation even in the presence of a relatively high screw.

The implantation level of 0.5 mm below the cartilage surface aimed at in this study was based on the biomechanical properties of talar cartilage (Li et al. 2008, Wan et al. 2008). Li et al. (2008) measured a peak contact strain of 30% (SD 6.1) after 30 s of loading, using magnetic resonance and dual-orthogonal fluoroscopic imaging. Similarly, Wan et al. (2008) measured a peak contact strain of 35% (SD 7.3) in human subjects with a mean medial talar dome cartilage thickness of 1.42 (SD 0.31) mm. To compensate for the compressible property of cartilage, the incompressible metallic implant was aimed 0.5 mm (i.e. 35% of 1.42 mm) below the cartilage surface in our study. The average implantation level achieved, 0.45 mm below the level of the articular cartilage, corresponds to 31% (95% CI: 23–40) of 1.42 mm.

In the event that the implant would be non-weight bearing due to deep implantation, the remaining weight-bearing area of the talar dome has been shown to be capable of carrying the loads without any statistically significant alteration in contact pressure (Christensen et al. 1994). In particular, the periphery of the implant cap should be recessed because “edge-loading” may result in a poor outcome (Koh et al. 2006, Becher et al. 2008). The lateral part of the implant can be considered to be crucial because it is located on the weight-bearing talar dome articulating with the tibial plafond. Here, the implant was recessed in all cases (mean 0.57 mm) with little variability (SD 0.16 mm). In contrast, the medial part of the implant is located on an area of the talus that typically bears less weight, i.e. the medial talar facet. Hence, a protruding implantation on the medial side is possibly less harmful to the opposite cartilage of the medial malleolus.

Our study has limitations. We used cadaver specimens of elderly individuals (aged 66–89 years), whereas patients suffering from an OD are often young adults. The cartilage of our specimens may have undergone degenerative changes with aging. The ankle joint is not, however, prone to osteoarthritic changes during aging (Cole et al. 2003) and there is no correlation between the thickness of talar cartilage and age (Shepherd and Seedhom 1999). Furthermore, the specimens in our study were macroscopically graded: only those with intact cartilage were included.

Contact pressures were measured under static loading, while dynamic or cyclic loading may be advocated because of its supposedly better representation of real life. We applied static loading because more cartilage deformation can be obtained by one continuous load than by short-time loads with intervals (Herberhold et al. 1999). Because there is more cartilage deformation with static loading, any excessive contact pressure due to elevated implantation might be detected more adequately. This implies that, if tested under dynamic loading, a higher implantation level might be erroneously accepted. To imitate the stance phase of gait, the joint was loaded in three positions that correspond to the mean sagittal ankle motion with corresponding forces (Stauffer et al. 1977).

The loads were applied to the tibia, as was done in previous studies (Ramsey and Hamilton 1976, Macko et al. 1991, Lloyd et al. 2006). The real-life situation might have been better imitated by also loading the fibula, which transmits 7% of the total force through the lower leg (Goh et al. 1992). However, loading the fibula would probably not have had any notable effect on the results because the pressure distribution would have shifted slightly to the lateral side (Thordarson et al. 1997), with minor effects on the medially-located implant.

Variability between specimens is a common finding in cadaver experiments (Matricali et al. 2009). The contact pressure of the prosthetic area before implantation ranged from 0.02% to 18% in the cadavers we used (Table 2). This variability does not affect the conclusions, as each talus served as its own control.

Possible inaccuracies of Tekscan pressure-sensitive sensors are their sensitivity to moisture and temperature, wrinkling, and change of their position during loading (Svoboda et al. 2002, Becher et al. 2008). By testing in a single lab, any inaccuracy due to difference in moisture or temperature was minimized. To detect inaccuracy due to wrinkling, the sensor was inspected after a loading sequence and checked under the lever arm loading system. It was replaced in one specimen. Changes in sensor position were minimized by taking digital photographs at the first testing sequence, which we used for verification of positioning at the second sequence.

Our findings form a first basis for clinical use of the implant, but its clinical effectiveness remains to be investigated. Other designs of this prosthetic implant have been used clinically for different joint surfaces, including the first metatarsal head (Hasselman and Shields 2008), the femoral head (van Stralen et al. 2009), the humeral head (Uribe and Botto-van Bemden 2009), and the patella (Davidson and Rivenburgh 2008). Although the short-term clinical outcome of these designs is promising, it remains to be investigated whether the implant is effective for the treatment of ODs of the talus, in terms of reducing pain and preventing cyst formation.

In conclusion, our study shows that accurate and reproducible implantation of this novel metallic implant can be achieved, preventing excessive prosthetic pressure. The results suggest that the implant can be used clinically in a safe way, but the effectiveness and safety of this treatment option should be evaluated in a clinical study.
